# Cathepsin B-associated Activation of Amyloidogenic Pathway in Murine Mucopolysaccharidosis Type I Brain Cortex

**DOI:** 10.3390/ijms21041459

**Published:** 2020-02-20

**Authors:** Gustavo Monteiro Viana, Esteban Alberto Gonzalez, Marcela Maciel Palacio Alvarez, Renan Pelluzzi Cavalheiro, Cinthia Castro do Nascimento, Guilherme Baldo, Vânia D’Almeida, Marcelo Andrade de Lima, Alexey V. Pshezhetsky, Helena Bonciani Nader

**Affiliations:** 1 Department of Biochemistry, Universidade Federal de São Paulo (UNIFESP), São Paulo, SP 04044-020, Brazil; mpalvarez.marcela@gmail.com (M.M.P.A.); rpcavalheiro@gmail.com (R.P.C.); hbnader@unifesp.br (H.B.N.); 2 Gene Therapy Center, Hospital de Clínicas de Porto Alegre, Porto Alegre, RS 90035-903, Brazil; esteban078@gmail.com (E.A.G.); gbaldo@hcpa.edu.br (G.B.); 3 Department of Psychobiology, Universidade Federal de São Paulo (UNIFESP), São Paulo, SP 04024-002, Brazil; cinthiaccn@gmail.com (C.C.d.N.); vaniadalmeida@uol.com.br (V.D.); 4 Molecular & Structural Biosciences, School of Life Sciences, Keele University, Huxley Building, Keele, Staffordshire ST5 5BG, UK; mlimagb@gmail.com; 5 Division of Medical Genetics, CHU Ste-Justine Research Centre, Montreal, QC H3T 1C5, Canada

**Keywords:** glycosaminoglycans, cathepsin B, Alzheimer’s disease, amyloid precursor protein, lysosomes, neuroinflammation

## Abstract

Mucopolysaccharidosis type I (MPS I) is caused by genetic deficiency of α-l-iduronidase and impairment of lysosomal catabolism of heparan sulfate and dermatan sulfate. In the brain, these substrates accumulate in the lysosomes of neurons and glial cells, leading to neuroinflammation and neurodegeneration. Their storage also affects lysosomal homeostasis-inducing activity of several lysosomal proteases including cathepsin B (CATB). In the central nervous system, increased CATB activity has been associated with the deposition of amyloid plaques due to an alternative pro-amyloidogenic processing of the amyloid precursor protein (APP), suggesting a potential role of this enzyme in the neuropathology of MPS I. In this study, we report elevated levels of protein expression and activity of CATB in cortex tissues of 6-month-old MPS I (*Idua* -/- mice. Besides, increased CATB leakage from lysosomes to the cytoplasm of *Idua* -/- cortical pyramidal neurons was indicative of damaged lysosomal membranes. The increased CATB activity coincided with an elevated level of the 16-kDa C-terminal APP fragment, which together with unchanged levels of β-secretase 1 was suggestive for the role of this enzyme in the amyloidogenic APP processing. Neuronal accumulation of Thioflavin-S-positive misfolded protein aggregates and drastically increased levels of neuroinflammatory glial fibrillary acidic protein (GFAP)-positive astrocytes and CD11b-positive activated microglia were observed in *Idua* -/- cortex by confocal fluorescent microscopy. Together, our results point to the existence of a novel CATB-associated alternative amyloidogenic pathway in MPS I brain induced by lysosomal storage and potentially leading to neurodegeneration.

## 1. Introduction

Mucopolysaccharidoses (MPS) are inherited metabolic diseases, caused by a deficiency of lysosomal enzymes involved in the degradation of glycosaminoglycans (GAGs). The main biochemical hallmark of these diseases is the intralysosomal accumulation of nondegraded or partially degraded GAGs, causing chronic and progressive deterioration of several tissues and organs leading eventually to multiple organ dysfunction [1].

Mucopolysaccharidosis type I, or MPS I, is caused by the deficiency of the lysosomal enzyme α-l-iduronidase (*IDUA*; E.C. 3.2.1.76) involved in lysosomal catabolism of two specific GAGs: heparan sulfate (HS) and dermatan sulfate (DS). This causes HS and DS storage in cells of different tissues (e.g., brain, liver, kidney, heart, and bones) and increases their levels in urine and blood. Main clinical manifestations include cardiorespiratory impairment, craniofacial dysmorphism, hepatosplenomegaly, joint stiffness, bone disorders and, in severe cases, neurological impairment [1,2,3,4,5,6].

The cellular pathology of MPS I is still under investigation, however, previous studies using animal models of the disease indicated that the impairment of GAG catabolism induces multiple pathological cascades in cells and tissues including calcium signaling defects [7,8,9,10], impaired bone turnover [11,12,13], inflammation, and defective vesicular trafficking [7,14,15]. Furthermore, impairment of autophagy in the neurons has been related to the defective turnover and storage of other metabolites such as cholesterol and gangliosides (e.g., GM2 and GM3). This has been proposed to cause an intracellular metabolic imbalance and, consequently, loss of neuronal viability [16,17,18].

Importantly, metabolic imbalance, neuroinflammation, and excessive neuronal death are also common features of adult neurodegenerative diseases including Alzheimer’s disease (AD). The main pathological hallmark of AD is the deposition of senile plaques, mainly constituted by aggregates of β-amyloid peptides in the patient’s brain thought to be the cause of massive neuroinflammation. Moreover, recent studies indicated that the formation of β-amyloid plaques and neurofibrillary tangles is also caused by defects in the autophagic-lysosomal pathway, essential for the removal of misfolded and aggregated proteins or damaged organelles [19,20,21,22,23]. In particular, accumulation of β-amyloid and phosphorylated Tau proteins have been reported in the mouse models of neurological MPS including MPS IIIB and MPS IIIC diseases [18,24,25,26,27,28].

The internalization and subsequent processing of the amyloid precursor protein (APP) by β-secretase 1 (BACE-1) and γ-secretase have been recognized as the main mechanism underlying generation of β-amyloid peptides [29,30,31]. However, other studies have demonstrated that additional lysosomal proteases such as cathepsin B (CATB) also play a role in amyloidogenic APP processing [32,33,34,35,36] and neuroinflammation [37,38,39]. Previously, we have demonstrated an increase of CATB activity in the tissues of MPS mouse models suggesting that these changes can increase amyloidogenesis and contribute to neuronal dysfunction and cognitive impairment [40,41,42]. In the current study, we demonstrate that lysosomal impairment in cortical neurons of MPS I mice is associated with overexpression of CATB, its subsequent leakage from lysosomes to the cytoplasm, accumulation of amyloid aggregates, and massive neuroinflammation.

## 2. Results

### 2.1. Altered Lysosomal Homeostasis and Amyloidogenic APP Processing in the Brain Cortex of Idua -/- Mice

To test our hypothesis that disruption of lysosomal homeostasis in brain cortex of MPS I (*Idua* -/-) mice leads to altered levels of lysosomal cathepsins and affects amyloidogenic APP processing, we studied brain tissues for the signs of lysosomal storage, neuroinflammation, and accumulation of amyloid aggregates. We have also measured protein levels and specific activity of CATB, which have been previously linked to the amyloid processing in Alzheimer’s disease animal models [33,43,44]. We decided to use 6-month-old *Idua -/-* mice because this is the age when symptoms of the CNS disease become evident [18,45,46,47].

**Figure 1:**
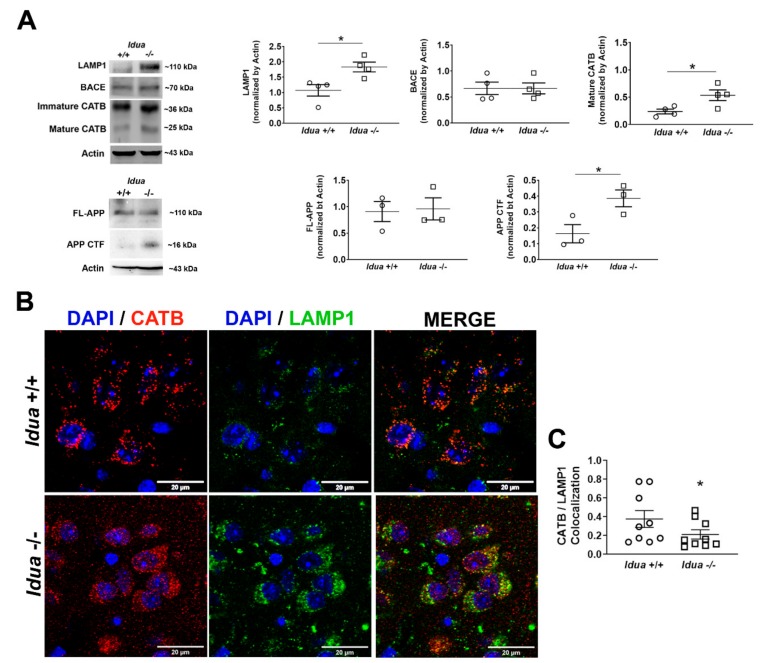
Alterations in lysosomal homeostasis and amyloidogenic amyloid precursor protein (APP) processing in *Idua* -/--/- mice cortex. (**A**) Representative Western blot images and quantitative analysis of band intensities (normalized by actin) for lysosomal-associated membrane protein 1 (LAMP1), β-secretase 1 (BACE-1), immature and mature forms of cathepsin B (CATB), full-length APP (FL-APP), and C-terminal fragment of APP (APP CTF) in brain cortex of 6-month-old *Idua* +/+ and *Idua* -/- mice. (**B**) Representative images of confocal laser scanning microscopy of 6-month-old *Idua* +/+ and *Idua* -/- brain cortex (layers IV-V) showing colocalization of CATB/LAMP1 (DAPI (4,6-diamidino-2-phenylindole dihydro-chloride) was used as the nuclear counterstain) in the cortical neurons. (**C**) Analysis of CATB/LAMP1 colocalization in the cortical neurons using the Manders’ coefficient. A total of 30 images were analyzed for each brain cortex. Data are expressed as the mean ± standard error mean of the mean (s.e.m) of three independent experiments for each animal. * *p* < 0.05 (Unpaired Student’s t test; *n* = 3–5 animals per genotype).

As indicated in Figure 1A, increased lysosomal-associated membrane protein 1 (LAMP1) levels were found in 6-month-old *Idua* -/- cortex when compared with *Idua* +/+, indicating increased lysosomal biogenesis and consistent with lysosomal storage cellular phenotype. While protein levels of the full-length APP (FL-APP) were similar in the cortex tissues from *Idua* +/+ and *Idua* -/- mice, significantly elevated levels of the ~16 kDa C-terminal APP fragment (Ab 1–40 and Ab 1–42 peptides together) were found in brain cortex from *Idua* -/- animals, indicative of enhanced amyloidogenic APP processing. The main pathway for production of this C-terminal APP fragment involves cleavage of APP by BACE-1; however, levels of BACE-1 protein were similar between *Idua* +/+ and *Idua* -/- groups (Figure 1A). On the other hand, the levels of the mature 25-kDa form of CATB were significantly elevated in the *Idua* -/- group prompting us to investigate the potential role of CATB in amyloidogenesis.

As shown in Figure 1B,C, a reduced colocalization between CATB and LAMP1 was observed in brain cortex of 6-month-old *Idua* -/- mice, suggestive of permeabilization of the lysosomal membrane with consequent leakage of CATB to the cytoplasm.

### 2.2. Increased CATB Activity in the Cortex from Idua -/- Mice

**Figure 2:**
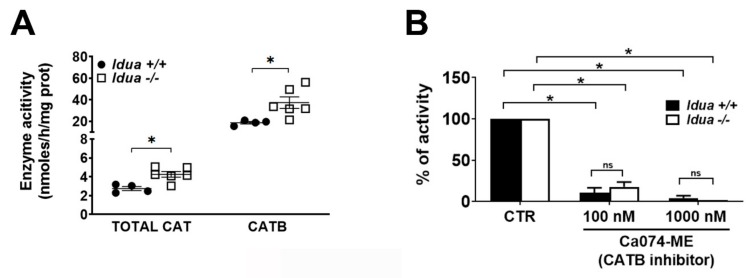
**Increased enzymatic activity of CATB in 6-month-old *Idua* -/- mice cortex.** (**A**) Pan-cathepsin and CATB enzymatic activity were measured using Z-Phe-Arg-AMC and Z-Arg-Arg-AMC substrates, respectively. (**B**) Inhibition of pan-cathepsin activity against Z-Phe-Arg-AMC by the specific CATB inhibitor CA074-ME at 100 nM and 1000 nM. Data are expressed as the mean ± s.e.m. of 3 independent experiments. (* *p* < 0.05; in unpaired Student’s t test; *n* = 3–5 mice per genotype).

Since GAG storage can affect activity of lysosomal enzymes, including cathepsins [48,49], we evaluated whether enzymatic activity of CATB in the cortex tissues from *Idua* -/- mice is increased similarly to its protein levels as detected by Western blots. Bulk cathepsin activity was measured in cortex homogenates using the substrate Z-Phe-Arg-AMC that is cleaved by CATB, CATK, and CATL, and the activity of CATB was determined using the specific substrate Z-Arg-Arg-AMC. A 1.5-fold increase in total cathepsin activity was observed in tissues from *Idua* -/- when compared with *Idua* +/+ mice (Figure 2A).

At the same time, brain tissues from *Idua* -/- mice presented an approximately 2-fold increase of specific CATB activity as compared with wild-type mice. The inhibition assay (Figure 2B), used to determine the fraction of specific CATB activity among others cathepsins (CATK and CATL) in cortex tissue, showed that the specific CATB inhibitor Ca074-ME at 100 nM (three-fold higher than its IC50) was able to suppress the majority of total cathepsin activity against Z-Phe-Arg-AMC in tissues from both *Idua* +/+ (~90% reduction) and *Idua* -/- mice (~80% reduction), indicating that CATB is the most prominent Z-Phe-Arg-converting enzyme in the mouse cortex (Figure 2B).

### 2.3. Neuroinflammation and Accumulation of Amyloid Aggregates in the Cortex from Idua -/- Mice

**Figure 3:**
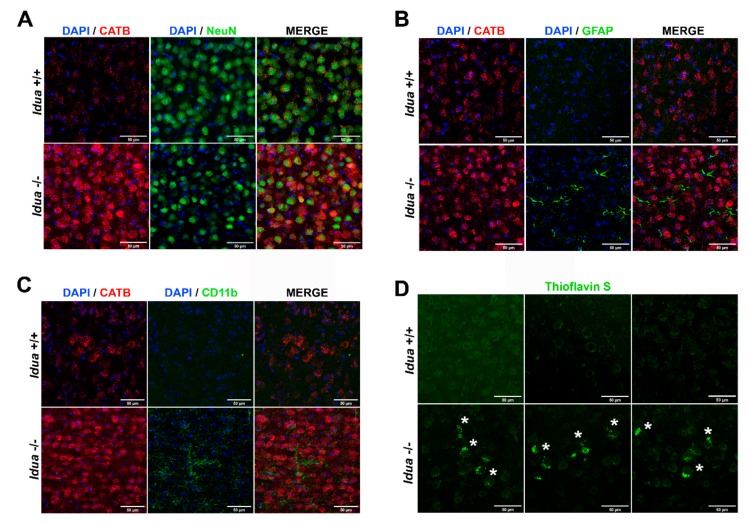
Increased levels of CATB, neuroinflammation, and Thioflavin S-positive aggregates in the brain cortex from *Idua* -/- mice. Representative images of confocal laser scanning microscopy of 6-month-old *Idua* +/+ and *Idua* -/- brain cortex immunostained for CATB and neuronal marker NeuN (**A**), CATB and astrocyte marker GFAP (**B**), and CATB and activated microglia marker CD11b (**C**). DAPI (4,6-diamidino-2-phenylindole dihydro-chloride) was used as a nuclear counterstain. (**D**) Thioflavin-S-positive protein aggregates suggestive of amyloid plaque deposition in 6-month-old *Idua* -/- brain cortex are indicated by white asterisks.

We further analyzed cortex tissues from MPS I and wild-type mice for the signs of neuroinflammation and accumulation of protein aggregates, the common hallmark of brain pathology in AD and neurological MPS. The brain slices were stained with antibodies against the neuronal marker protein NeuN (Figure 3A), the marker protein for astrocytes GFAP (glial fibrillary acidic protein; Figure 3B), and the marker protein for activated microglia CD11b (Figure 3C). The slices were counterstained with antibodies against CATB to determine which cells are mainly responsible for the increase of mature CATB protein in the cortex. The data demonstrate that NeuN-positive neurons expressed high levels of CATB that also showed a diffused pattern consistent with its cytoplasmic localization.

The cortices of *Idua* -/- mice, but not of the wild-type mice, contained high levels of GFAP- and CD11b-positive cells (Figure 3B and 3C, respectively), indicative of an increased number of activated astrocytes and microglia, the clear sign of neuroinflammation. Both astrocytes and microglia were mainly negative for CATB staining suggesting that it is expressed by pyramidal cortical neurons. Neurons in the MPS I mouse cortices also showed increased fluorescence staining with Thioflavin S (Figure 3D). This dye binds to beta sheet-rich structures and displays enhanced fluorescence, which is useful for the detection of misfolded proteins and specifically amyloid aggregates in the brains of AD patients. The increased Thioflavin staining is, therefore, suggestive of amyloid accumulation in the cortex of 6-month-old *Idua* -/- brain mice.

## 3. Discussion

Neurological impairment and progressive loss of cognitive functions are hallmarks of severe cases of MPS I, but the molecular mechanisms and cellular events responsible for these symptoms are not fully understood [2,3,18]. Previous studies using animal models of MPS I and other neurological MPS diseases revealed several affected pathways including dysfunctional GAG degradation/biosynthesis, altered lipid metabolism, induced inflammation, and autophagy block. Storage of undegraded lysosomal substrates severely affects the homeostasis of endo-lysosomal pathway leading to increased size and number of lysosomes, as well as variations in their intracellular position, movement, and docking with other vesicles. It also causes changes in the lipid composition and loss of integrity of the lysosomal membrane and alters expression and activity of lysosomal hydrolases in all affected cells and tissues, including the brain [11,13,16,40,50,51,52,53,54]. Nondividing cells such as brain neurons use autophagy as the main mechanism for clearance of protein aggregates and damaged organelles [55,56]. Lysosomal accumulation of nondegraded substrates can affect normal autophagy processes in these cells, leading to reduced clearance of misfolded intracellular proteins and accumulation of toxic protein aggregates in brain parenchyma, loss of neuronal viability, and neurodegeneration [7,20,57,58].

Previously, we have observed higher levels of LAMP1, CATB, and CATD but no loss in cell viability in murine MPS I hippocampus, indicating that decrease in behavioral manifestations such as reduced spatial awareness and memory is caused by neuronal dysfunction rather than neuronal death [40]. Higher LAMP1 levels, observed in cortex of 6-month-old *Idua* -/- mice, were indicative of increased number and size of lysosomes, both promoted in response to lysosomal storage of undegraded GAGs and other molecules [59]. In a similar fashion, Medina et al. [60] observed increased LAMP1 and intracellular Ca^2+^ levels in embryonic fibroblasts and neuronal stem cells overexpressing the transcription factor EB (TFEB), which resulted in increased exocytosis of the lysosomal content. The current study demonstrates increased levels of mature CATB protein and enzymatic activity in the brain cortex tissues of 6-month-old MPS I mice. Elevated CATB immunoreactivity was found primarily in the pyramidal cortical neurons (layers 4–5) that show lysosomal storage phenotype and contain Thioflavin S-positive misfolded protein aggregates. Importantly, confocal microscopy revealed that the intracellular localization of CATB in the *Idua* -/- neurons is drastically different from that in the wild-type cells. While in cells of normal control mice CATB is associated with perinuclear puncta positive for lysosomal marker LAMP1, in the *Idua* -/- neurons it shows diffused cytoplasmic staining and does not colocalize with LAMP1. All this is consistent with a suggestion that CATB leaks from lysosomes due to the loss of integrity of the lysosomal membrane associated with intralysosomal accumulation of undegraded GAGs.

By itself, permeabilization of the lysosomal membrane and leakage of lysosomal proteases such as CATB into the cytoplasm has been shown to induce a complex and sequential activation of cell death effectors, leading to apoptosis [61,62], so it is tempting to speculate that it can be an important contributing factor to neuronal loss in the MPS I brain. Besides, our data suggest that the massive induction of the cytoplasmic pool of CATB can be potentially linked to another pathological pathway leading to neurodegeneration by participation in the amyloidogenic processing of APP. We show that while the level of full-length APP is not increased in the cortex tissues of *Idua -/-* mice, the amount of processed 16-kDa CTF APP peptide, also called C-terminal β-secretase fragment (CTFβ), is increased at least 2-fold. Importantly, the increased levels of CTFβ capable to form amyloid plaques [29] were coinciding with the appearance of misfolded protein aggregates in the cortical neurons.

Although further studies are necessary to establish a causative relationship between the induction of CATB levels in neuronal cytoplasm and amyloidogenic APP processing, several lines of evidence support this hypothesis. First, levels of BACE-1, major APP processing enzyme, do not differ between *Idua -/-* and *Idua +/+* brains suggesting that an increase in CTF generation occurs due to induction of another protease. Second, CATB has full enzymatic activity at the neutral pH of the cytoplasm and is protected from alkaline pH-induced inactivation by interaction with GAGs related to the stored HS, which can also enhance endopeptidase activity of this enzyme [63]. Elevated activity of lysosomal cathepsins and, in particular, that of CATB, observed in several lysosomal diseases other than MPS, can be also explained by the nuclear translocation of TFEB that promotes expression of several hundred genes related to lysosomal catabolism and autophagy (so-called CLEAR network), including *CTSB* encoding for CATB [64]. Most of pan-cathepsin activity (cathepsins B, K, and L together) elevated in 6-month-old *Idua* -/- mice has been blocked by the specific CATB inhibitor, which indicates that in the mouse cortex CATB is the most prominent among these cathepsins.

Previous studies have established that CATB is induced in the mouse model of AD expressing human mutant APP variant and plays a crucial role in amyloidogenesis observed in these animals [20,65]. When CATB in the brain of AD mice was depleted genetically or inhibited pharmacologically by administration of cysteine protease inhibitor E64d, the amyloid deposition and cognitive decline were delayed, suggesting that the enzyme may become a potential drug target for AD [34,36,43,66,67,68]. In contrast, inhibition of BACE-1 did not reduce production of CTFβ in the AD mouse brain or improve memory deficits [43,44].

While accumulation of protein aggregates including beta-amyloid is not the only pathophysiological mechanism causing neurodegeneration in the *Idua* -/- mice, this process coincides with massive neuronal death during the terminal acute phase of the disease [16]. Besides, it has been identified as a trigger of neuroinflammation [27]. In our study, confocal immunofluorescent microscopy revealed the presence of activated astrocytes (astrogliosis) and microglia (microgliosis) in 6-month-old *Idua* -/- cortex, which prompted us to speculate that increased CATB activity, amyloidogenic APP processing, neuroinflammation, and amyloid plaque deposition in *Idua* -/- brain cortex at advanced stages of the disease could be interconnected. We also found accumulation of intracellular amyloid aggregates and high levels of astromicroglyosis in the cortexes of human postmortem brains from patients with different neurologic MPS subtypes [69], suggesting that this pathogenic cascade can be a common neuropathological feature.

In conclusion, these data suggest that CATB-associated amyloidogenic APP processing leading to amyloid plaque formation in AD may also be present in MPS I and perhaps in other lysosomal neurodegenerative diseases. If such connection is proven, the use of CATB inhibitors coupled with the other treatments currently available for MPS I, such as hematopoietic stem cell transplantation and enzyme replacement, may be proposed to improve the therapeutic outcome.

## 4. Materials and Methods

### 4.1. Ethical Statement

All animal procedures were performed according to the Animal Research: Reporting of In Vivo Experiments (ARRIVE) guidelines for the care and use of animals [70]. All experiments were conducted in accordance with the Brazilian Federal Law for Animal Experiments and were approved by the Animal Care and Ethics Committee of the Universidade Federal de São Paulo (CEUA/UNIFESP # 5235140717). The animal experimentation conducted in Canada has been approved by the Animal Care and Use Committee of the Ste-Justine Hospital Research Center.

### 4.2. Mice

The murine MPS I model used in this work (*Idua* -/-) mice was generated on a C57BL/6 (*Idua* +/+) background as previously described [16] and was kindly donated by Dr. Elizabeth Neufeld (UCLA, USA) and Dr. Nance B. Nardi (ULBRA, Brazil) or obtained from The Jackson Laboratory (Bar Harbor, ME USA). *Idua* +/+ and *Idua* -/- colonies were established after heterozygous mating and maintained on a light-dark 12:12 cycle under controlled temperature conditions (20 ± 2 °C) with free access to food and water. After weaning, 6-month-old mice were genotyped by regular polymerase chain reaction using oligonucleotides that amplify a specific fragment of *Idua* gene [71]. After euthanasia, brains were removed and stored at −80 °C for biochemical analyses or fixed in buffered paraformaldehyde (PFA) solution for immunofluorescence analyses.

### 4.3. Immunofluorescence and Confocal Microscopy

Fixed brains were embedded in TissueTek^®^ optimum cutting temperature (O.C.T.) compound (Sakura, USA) and cut into sequential 10 µm thick sagittal cross-sections by a Leica CM1950 Cryostat (Leica Microsystems, Wetzlar, Germany) for immunofluorescence analysis. Brain slices from *Idua* +/+ and *Idua* -/- mice were post-fixed in 4% PFA for 30 min, washed with 0.1 M glycine, and blocked with 5% bovine serum albumin (BSA) for 1 h. Slices were then incubated with the following primary antibodies: rabbit anti-cathepsin B (1:200; Calbiochem, La Jolla, California, USA; catalog number: 219408); mouse anti-LAMP1/1D4B (1:200; 1D4B was deposited to the Developmental Studies Hybridoma Bank by August, J.T); rabbit anti-GFAP/8-1E7 (1:200; 8-1E7 was deposited to the Developmental Studies Hybridoma Bank by De Blas, Angel L.); rat anti-CD11b (1:50; M1/70.15.11.5.2 was deposited to the Developmental Studies Hybridoma Bank by Springer, T.A.); mouse anti-NeuN (1:200; Biolegend, San Diego, California, USA; catalog number: SIG-39860) in 0.1% BSA / 0.3% Triton X-100 at 4 °C in a light-protected humidity chamber for 16 h. Secondary antibodies (all purchased from Thermo Fischer Scientific, Waltham, MA, USA) were Alexa Fluor^®^ 488-conjugated donkey anti-mouse IgG (1:350; catalog number: A21202), Alexa Fluor^®^ 555-conjugated donkey anti-rabbit IgG (1:350; catalog number: A21428), and Alexa Fluor^®^ 633-conjugated goat anti-rat IgG (1:350; catalog number: A21094). DAPI (4,6-diamidino-2-phenylindole dihydro-chloride) was used for nuclear staining (1:10,000; Thermo Fisher Scientific, Waltham, MA, USA; catalog number: D1306). To detect misfolded protein aggregates, brain slices were washed with phosphate buffered solution (PBS), incubated with 0.3% Triton X-100 in PBS for 1 h at RT, and stained with 0.05% Thioflavin-S (Sigma, USA; catalog number: T1892) in PBS in the dark for 15 min, washed in 50% ethanol twice for 1 min and, then, in water for 3 min. Thioflavin-S fluorescence was analyzed using the green channel (excitation: 488 nm/emission: 510–560 nm). Glass coverslips (Dako, DK; catalog number: CS704) were mounted on glass microscope slides using Fluoromount-G mounting medium (Southern Biotech, USA; catalog number: 0100-01) and representative images from the whole tissue were captured with a Leica TCS SP8 Confocal Laser Scanning Microscope (Leica Microsystems, DE). Fluorescence observation was made through oil-immersion Plan-Apochromat 20× or 63× objectives (numerical aperture 1.4). Images were represented as maximum intensity projections corresponding to the z-series of all confocal stacks. Images were mounted using the ImageJ software (US National Institutes of Health, Bethesda, MD, USA). Colocalization was analyzed using the corresponding tool from Leica LAS-AF image analysis software. After setting adequate background and threshold, images were overlaid revealing the colocalized voxels. The percentage of colocalization was calculated according to Manders’ overlap coefficient, which has values between 0 and 1. The closer the value is to 1, the stronger the colocalization.

### 4.4. Western Blotting

After dissection, brain cortex from 6-month-old *Idua* +/+ and *Idua* -/- mice were homogenized using a glass dounce homogenizer in ice-cold non-denaturing lysis buffer (150 mM NaCl, 1% NP-40, 50 mM Tris, pH 8.0) supplemented with 10% of protease inhibitor cocktail (Roche, New York, NY, USA). After clearing by centrifugation at 12,000× *g* for 20 min, total protein concentration was measured using the Pierce BCA Protein Assay Kit (Thermo Fisher Scientific, Waltham, Massachusetts, USA). Equal aliquots were stored at −80 °C for subsequent analysis by co-immunoprecipitation and Western blotting. For blotting analyses, an equal volume of sodium dodecyl sulfate (SDS) gel loading buffer was added and the samples incubated in a boiling bath for 10 min. Protein extracts (50 μg) were resolved by SDS–PAGE on 10%–15% gels (29:1 acrylamide:bis-acrylamide) and transferred to nitrocellulose membranes (Millipore, Danvers, MA, USA). Membranes were blocked with 5% BSA in TBS-T (Tris-buffered saline with 0.1% Tween-20, pH 8.4) for 1 h at room temperature and then incubated overnight at 4 °C with the following primary antibodies (all diluted 1:1000 in TBS-T/5% BSA): rabbit anti-APP (Cell Signaling Technologies, Danvers, MA, USA; catalog number 2452), rabbit anti-BACE-1 (Cell Signaling Technologies, Danvers, MA, USA; catalog number 5606), rabbit anti-cathepsin B (Calbiochem, La Jolla, CA, USA; catalog number 219408); rabbit anti-LAMP1 (Abcam, UK; catalog number 24170), and goat anti-actin I-19 (Santa Cruz Biotechnologies, Santa Cruz, CA, USA; catalog number B1116). After several washes with TBS–T, membranes were subsequently incubated with the following secondary antibodies: ECL^TM^ donkey anti-rabbit IgG, horseradish peroxidase linked (1:2500; GE Healthcare, Chicago, IL, USA; catalog number NA934V) and rabbit anti-goat IgG, peroxidase conjugate (1:10,000; Merck, West Point, PA, USA; catalog number A-8919), accordingly. The immunoblots were revealed by chemiluminescence with SuperSignal West Pico PLUS (Thermo Fisher Scientific, Waltham, MA, USA). Total actin protein was used for signal normalization. Bands were acquired using a gel documentation system (M-ChemBIS; DNR Bio-imaging Systems, ISR) and the intensities quantified using the TotalLab TL100 v2006 one-dimensional gel analysis software (Nonlinear Dynamics, UK).

### 4.5. Enzyme Assays

Brain cortex tissues from *Idua* +/+ and *Idua* -/- mice were homogenized in acetate buffer (100 mM sodium acetate, 2.5 mM EDTA, 0.01% Triton X-100, 2.5 mM dithiothreitol, pH 7.4), aliquoted and stored at −80 °C for subsequent enzymatic assays. Total activity of CATB, cathepsin K (CATK), and cathepsin L (CATL) was measured using 10 μM of the substrate, benzyloxycarbonyl-l-phenylalanyl-l-arginine-7-amido-4-methylcoumarin (Z-Phe-Arg-AMC; AnaSpec, Fremont, CA, USA) in the same acetate buffer. The reaction mixture was placed in a well of a microtiter plate and incubated at 37 °C. Fluorescence was read on the instrument Flexstation 3 multi-mode microplate reader (Molecular Devices, San Jose, CA, USA), using the wavelengths of 355 nm (excitation) and 460 nm (emission). Readings were taken every 10 min for a total of 40 min. For CATB activity, the specific substrate Z-Arg-Arg-AMC (Enzo Life Sciences, Farmingdale, NY, USA) was used at a final concentration of 100 μM, with the same buffer and parameters for the total cathepsins assay. The product concentrations were calculated using a calibration curve established with a standard 7-amino-4-methylcourmarin (AMC; AnaSpec, Fremont, CA, USA). The results were expressed as nmol/h/mg protein. For the inhibition assay, the specific CATB inhibitor Ca074-ME (Calbiochem, La Jolla, CA, USA; catalog number 219385,) was added to tissue homogenate at a final concentration of 100 nM or 1000 nM, as indicated, prior to the incubation with total cathepsin substrate (Z-Phe-Arg-AMC; Enzo Life Sciences, Farmingdale, NY, USA) at pH 7.4. Inhibition results were expressed as a percentage of total cathepsin activity in the absence of the inhibitor.

### 4.6. Statistics

All data were expressed as mean ± standard error mean of the mean (s.e.m.). After verifying that the data showed normal distribution, they were analyzed by unpaired Student’s *t* test (for single comparisons) and ANOVA (for multiple comparisons) using GraphPad Prism 5 for Windows (GraphPad Software, La Jolla, CA, USA). The level of significance was set at *p* < 0.05.
